# Unmasking Euglycemic Diabetic Ketoacidosis: The Interplay of Pregnancy, Sepsis, and Glucagon-Like Peptide 1 Analog

**DOI:** 10.7759/cureus.78193

**Published:** 2025-01-29

**Authors:** Alice Huang, Jenny Lu, Nicole Pancotto, Gagandeep S Sekhon, Isabel M Gossen, July M Reyes, Roxana Lazarescu

**Affiliations:** 1 Internal Medicine, Touro College of Osteopathic Medicine, New York, USA; 2 Medicine, Touro College of Osteopathic Medicine, New York, USA; 3 Internal Medicine, Xavier University School of Medicine, Oranjestad, ABW; 4 Internal Medicine, St. George’s University School of Medicine, West Indies, GRD; 5 Internal Medicine, Wyckoff Heights Medical Center, New York, USA

**Keywords:** diabetes type 1, diabetes type 2, edka, glp-1 ra, glp-1 receptor agonist

## Abstract

Euglycemic diabetic ketoacidosis (EDKA) is a life-threatening, yet under-recognized, complication of type 1 and type 2 diabetes. EDKA is characterized by metabolic acidosis and ketonemia in the absence of significant hyperglycemia. The absence of ketoacidosis-related complications may delay diagnosis, leading to severe consequences. Common precipitating factors include medication use (e.g., sodium-glucose cotransporter 2 inhibitors), acute illnesses (e.g., sepsis), trauma, and pregnancy.

We report the case of a 35-year-old pregnant female with a history of type 2 diabetes, hypertension, and hyperlipidemia who presented with a two-day history of chills, shoulder pain, and reduced range of motion. The patient was compliant with dulaglutide, but not insulin. She had a history of chronic left shoulder pain managed with intraarticular corticosteroid injections, and imaging showed no evidence of fracture. The patient was admitted for septic arthritis of the left shoulder. During her hospital stay, she developed an elevated anion gap metabolic acidosis with a small elevation in her blood glucose. She tested positive for ketonuria and glucosuria. Upon admission, she deteriorated and was transferred to the intensive care unit for the management of EDKA. An insulin drip was initiated, leading to the resolution of her anion gap and resolving the ketoacidosis.

This case underscores the importance of recognizing EDKA, particularly in the context of rising glucagon-like peptide 1 receptor agonist use for diabetes and weight management. EDKA remains a rare but serious condition, and timely identification and management are critical in preventing morbidity. This report also emphasizes the need for heightened clinical suspicion of EDKA in pregnant or septic patients with diabetes, even when blood glucose levels do not suggest diabetic ketoacidosis.

## Introduction

Euglycemic diabetic ketoacidosis (EDKA) is a life-threatening emergency. However, it is an under-recognized complication of type 1 and type 2 diabetes. EDKA is classified as having metabolic acidosis (pH: <7.3, serum bicarbonate: <18 mmol/L) and ketonemia without hyperglycemia (blood glucose: <250 mg/dL) [1]. The absence of hyperglycemia may delay diagnosis inducing severe consequences. Factors that may precipitate EDKA in type 2 diabetes are medication-induced (sodium-glucose cotransporter 2 (SGLT2) inhibitors), acute-onset illness (sepsis, trauma), and pregnancy [1]. In this report, we highlight a case that has the interplay of pregnancy and sepsis of the shoulder in a pregnant woman who was taking a glucagon-like peptide 1 (GLP-1) analog and was non-compliant with insulin therapy.

## Case presentation

A 35-year-old pregnant female with a past medical history of hypertension, hyperlipidemia, and type 2 diabetes presented with a two-day history of chills and left shoulder pain with decreased range of motion. There was no evidence of a fracture upon previous imaging. The patient had a history of chronic left shoulder pain managed with intra-articular therapeutic injections at the pain management clinic. Following a routine visit to the pain management facility, she developed increased pain in her left shoulder which quickly led to admission for suspicion of septic arthritis.

Investigation

**Figure 1:**
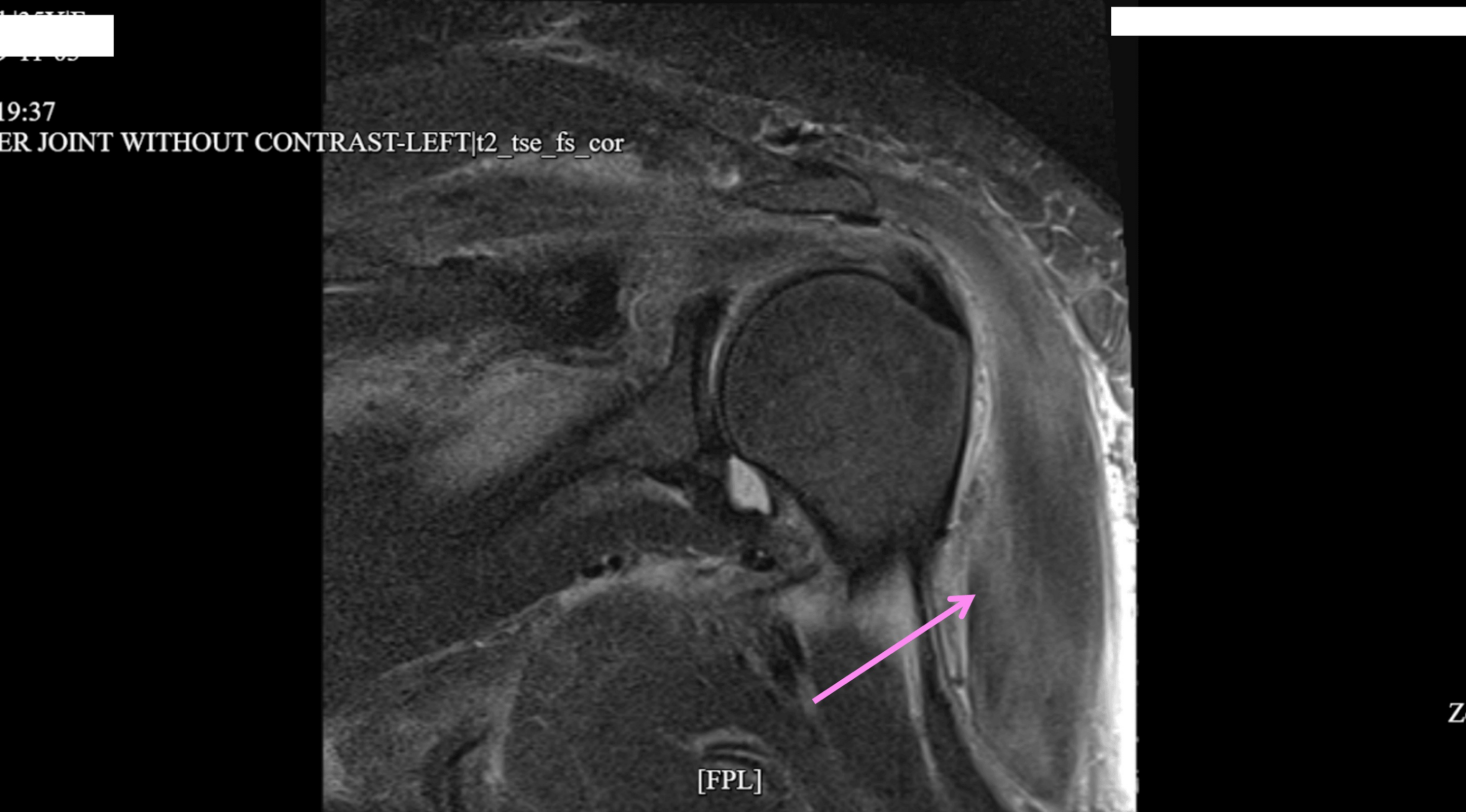
Magnetic resonance imaging without contrast showing diffuse cellulitis and myositis of the left deltoid.

**Figure 2:**
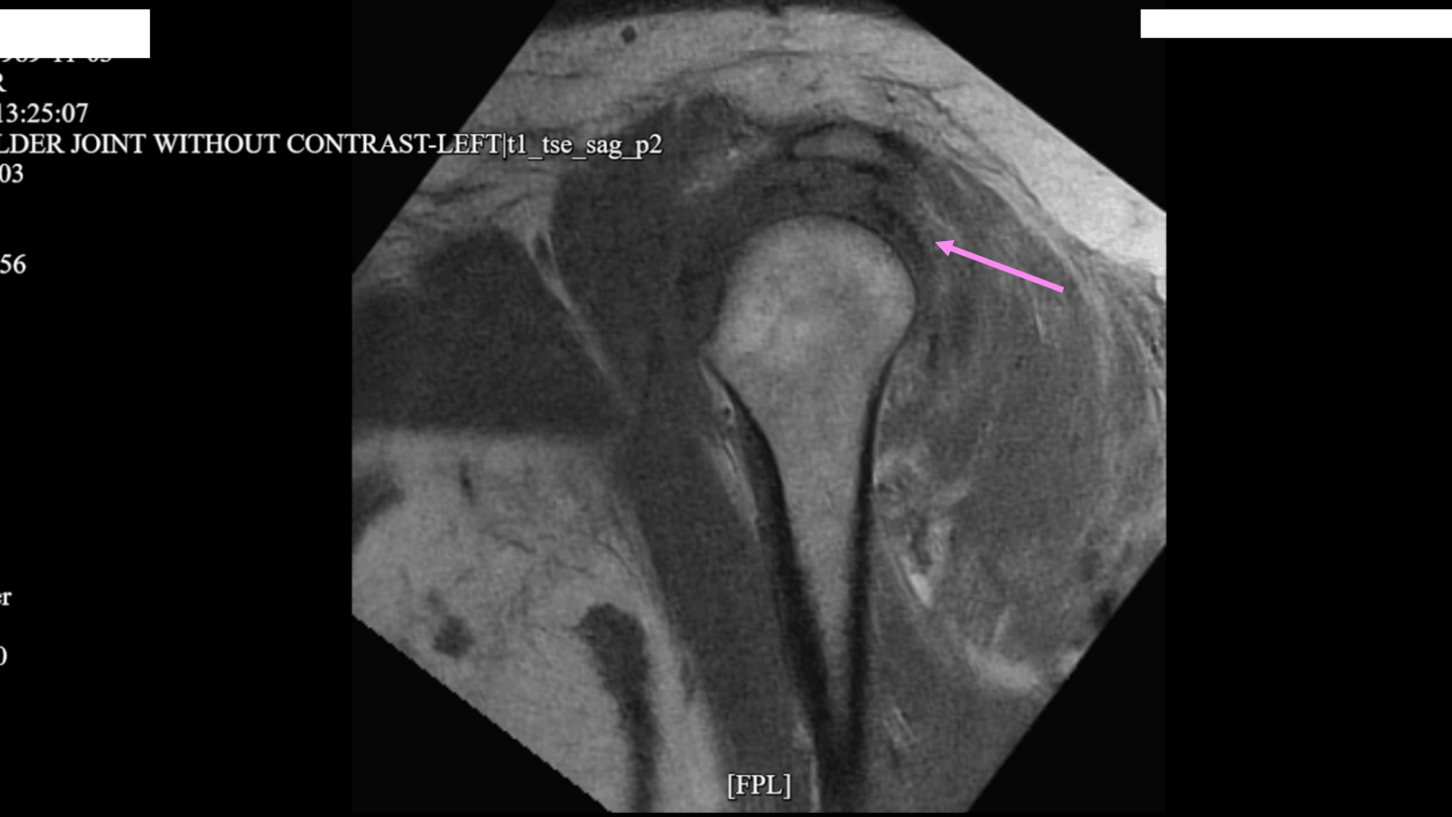
Magnetic resonance imaging without contrast showing small glenohumeral joint effusion on the left upper extremity.

**Figure 3:**
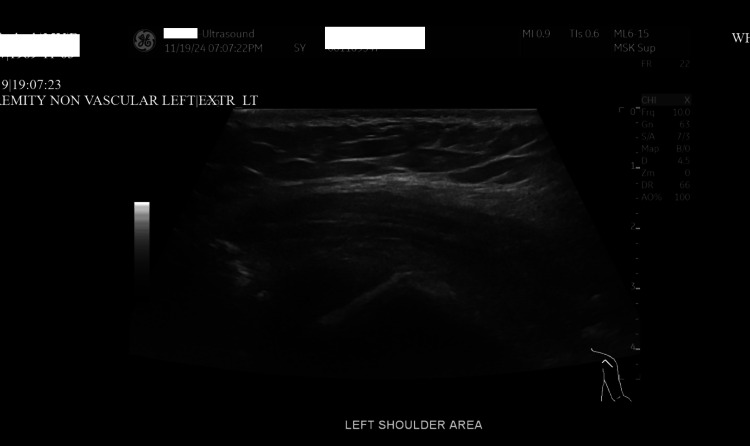
Non-vascular ultrasound of the left upper extremity shows no evidence of soft tissue collection or other abnormalities.

Figures 1-3 show diffuse cellulitis, myositis, and effusion of the left deltoid with no soft tissue collection. The patient who was admitted for septic arthritis after meeting sepsis criteria underwent orthopedic surgery including arthroscopic irrigation and debridement and subacromial decompression of the left shoulder. The wound culture showed growth of *Staphylococcus aureus* which was resistant to clindamycin, but sensitive to oxacillin. Infectious disease consult recommended vancomycin and ceftriaxone for the treatment of sepsis. However, the patient was sent to the intensive care unit (ICU) upon discovery of high anion gap metabolic acidosis (arterial blood gas: pH = 7.30, O_2_ = 24 mmHg, HCO_3_ = 11.8 mEq/L) with a glucose level of 250 mg/dL. The patient had been compliant with taking dulaglutide; however, she was non-compliant with taking her insulin before admission. Upon arrival at the ICU, the patient was put on an insulin drip and intravenous (IV) fluids with tight monitoring until the high anion gap was closed. The patient was switched to IV cefazolin, as recommended by infectious disease. Her high anion gap was closed and she was transferred to the medical ICU where she was transitioned to long-acting insulin.

Treatment and management

The patient was initially treated with vancomycin and ceftriaxone for septic arthritis of the shoulder, which was later adjusted to cefazolin following joint aspiration. The potential adverse effects of cefazolin (altered fetal microbiome development, gastrointestinal disturbances, and maternal allergies) were discussed with the patient, but she opted to continue with the treatment as she was planning on terminating her pregnancy.

The patient was initially placed on an insulin drip and subsequently transitioned to subcutaneous insulin (Glargine at bedtime and Lispro before meals). Blood glucose was monitored every four hours, and fluid resuscitation was provided. Her hypertension was managed with blood pressure monitoring and adjustments to antihypertensive therapy as needed. The pain was effectively managed and reduced.

Outcome and follow-up

The patient’s condition improved significantly with medical management, achieving stabilization with improved blood glucose control, and management of active septic arthritis and diabetic ketoacidosis. She was also educated on the importance of insulin adherence. Outpatient follow-up appointments for shoulder rehabilitation, infectious disease, endocrinology, gynecology, and primary care were also recommended.

## Discussion

EDKA is a rare but life-threatening complication that can occur in type 1 and type 2 diabetes. It is characterized by metabolic acidosis, decreased serum bicarbonate levels, and the presence of ketones in both serum and urine in the setting of blood glucose levels <250 mg/dL. Dulaglutide, a GLP-1 receptor agonist (GLP-1 RA), is a medication used to manage diabetes. It mimics the effects of GLP-1, promoting increased insulin secretion and reducing glucagon release, which helps lower blood glucose [2]. SGLT2 inhibitors are another class of diabetes medications that lower blood glucose.

While SGLT2 inhibitors have been implicated in cases of EDKA, the role of GLP-1 RAs in causing EDKA remains unclear [3]. Studies have not shown GLP-1 RAs to independently contribute to EDKA, and they are generally considered to have a low risk of hypoglycemia [4]. The mechanism of SGLT2 inhibitor-induced EDKA is theorized to involve insufficient insulin production due to lowered blood glucose, which may be inadequate to prevent lipolysis and hepatic ketogenesis [5]. Additionally, SGLT2 inhibitors increase the reabsorption of acetoacetate in the renal tubules, contributing to elevated ketone levels in the blood. Physicians should remain vigilant and monitor for signs of EDKA even if the patient’s blood glucose level is normal. They can also educate patients on symptoms of EDKA such as nausea, vomiting, and abdominal pain.

## Conclusions

Insulin non-compliance has been identified as a significant risk factor for SGLT2 inhibitor-induced EDKA. Given the increasing use of GLP-1 RAs in diabetic patients, further research is needed to assess whether the blood glucose-lowering effects of GLP-1 RAs could pose a similar risk for EDKA as observed with SGLT2 inhibitors. Clinicians should remain vigilant for the potential development of EDKA, particularly with the growing use of GLP-1 RAs.
